# CBCT-Guided Online Adaptive Ultrahypofractionated Whole-Breast Radiotherapy for Early-Stage Breast Cancer: Dosimetric Outcomes and Acute Toxicity

**DOI:** 10.3390/curroncol33100634

**Published:** 2026-10-20

**Authors:** Giovanni Ursi, Matea Lekić, Hrvoje Kaučić, Aleksandar Kovačević, Domagoj Kosmina, Karla Schwarz, Dragan Schwarz, Damir Vučinić

**Affiliations:** 1 Specialty Hospital Radiochirurgia Zagreb, 10431 Sveta Nedelja, Croatia; giovanni.ursi@radiochirurgia.hr (G.U.); matea.lekic@radiochirurgia.hr (M.L.); hrvoje.kaucic@radiochirurgia.hr (H.K.); aleksandar.kovacevic@radiochirurgia.hr (A.K.); domagoj.kosmina@radiochirurgia.hr (D.K.); karla.schwarz@radiochirurgia.hr (K.S.); dragan.schwarz@radiochirurgia.hr (D.S.); 2 Department of Physics, Faculty of Science, University of Zagreb, 10000 Zagreb, Croatia; 3 Medical Faculty Osijek, University Josip Juraj Strossmayer in Osijek, 31000 Osijek, Croatia; 4 Medical Faculty, Juraj Dobrila University of Pula, 52100 Pula, Croatia

**Keywords:** adaptive radiotherapy, breast neoplasms, radiotherapy, image-guided

## Abstract

Ultrahypofractionated whole-breast radiotherapy reduces treatment burden but increases the importance of accurate dose delivery at each fraction. This retrospective single-center study evaluated cone-beam computed tomography-guided online adaptive radiotherapy in 55 patients with early-stage breast cancer treated with 26 or 27 Gy in five fractions. Dosimetric analysis was available for 52 patients and 259 paired scheduled and adapted fractions. Linear mixed-effects models with a patient-specific random intercept were used to account for repeated fractions within individual patients. The adapted plan was selected for 242 out of 260 delivered fractions (93.1%). Mean planning target volume V95% increased from 93.52 ± 2.94% with the scheduled plan to 98.13 ± 1.04% with adaptation (*p* < 0.001), with reduced interfraction variability. Ipsilateral lung V30% increased modestly from 13.94% to 14.96% (*p* < 0.001). In left-sided cases, no significant differences were observed for heart V25% (1.400% vs. 1.395%; *p* = 0.3825) or left anterior descending coronary artery D0.03 cm^3^ (1.771 vs. 1.873 Gy; *p* = 0.1390). Acute grade 1 toxicity occurred in five patients (9.1%), with no grade ≥ 2 events. Mean treatment-session duration was 28 min. Online adaptation significantly improved daily target coverage and was technically feasible within a five-fraction whole-breast workflow. Acute toxicity was limited in this cohort, while longer follow-up and cumulative dose analyses are needed to further define its impact on organ-at-risk exposure and clinical outcomes.

## Simple Summary

Adjuvant radiotherapy after breast-conserving surgery is highly effective, but daily changes in breast shape, breathing, and internal anatomy can make it difficult to reproduce the exact conditions used for treatment planning. Online adaptive radiotherapy allows the treatment plan to be recalculated, and when needed, reoptimized immediately before each fraction based on the patient’s anatomy that day. In this single-center study of 55 patients with early-stage breast cancer treated in five fractions, daily adaptation consistently improved breast target coverage while maintaining acceptable doses to the heart and coronary artery, with only a small increase in lung exposure. Acute side effects were uncommon and mild. These findings support daily adaptation as a promising way to improve the robustness of five-fraction breast radiotherapy, particularly when breath-hold anatomy varies between treatment days.

## 1. Introduction

Adjuvant whole-breast radiotherapy remains an integral component of breast-conserving treatment for patients with early-stage breast cancer. Over the past decade, treatment schedules have progressively shifted toward shorter fractionation regimens, reducing the burden of radiotherapy without compromising oncological outcomes. The FAST-Forward randomized phase III trial established 26 Gy in five fractions over one week as a non-inferior alternative to 40 Gy in 15 fractions for local tumor control, with comparable normal-tissue effects at five years [1]. More recent prospective real-world data further support the clinical safety of the five-fraction approach, showing comparable or lower acute toxicity and similar patient-reported outcomes compared with 15-fraction treatment [2]. However, because treatment is condensed into only five fractions, each fraction represents a substantially greater proportion of the total treatment course, increasing the practical importance of accurate target coverage and organ-at-risk (OAR) sparing at every treatment session.

Breast radiotherapy is highly sensitive to day-to-day geometric variability. Changes in breast contour, postoperative edema, seroma, arm and shoulder position, thoracic configuration, and respiratory state can alter the spatial relationship between the target volume and surrounding OARs. Contemporary ESTRO-ACROP recommendations therefore emphasize reproducible positioning, immobilization, image guidance, and respiratory motion management as essential components of modern breast radiotherapy [3]. Deep-inspiration breath-hold (DIBH) has become particularly important in left-sided breast irradiation because inspiration increases the distance between the anterior heart and chest wall, reducing cardiac exposure [4,5]. Surface-guided radiation therapy (SGRT) further improves patient positioning and enables continuous, non-ionizing monitoring of the external surface and breath-hold stability. Nevertheless, reproducibility of the external thoracic surface does not necessarily imply identical internal anatomy at every fraction. Studies incorporating cone beam CT (CBCT) during breast radiotherapy have demonstrated residual interfraction variations in target and OAR geometry despite apparently stable surface-guided positioning and breath-hold performance [5,6].

This distinction is particularly relevant to the conventional DIBH workflow. A non-adaptive treatment plan is optimized using the anatomy obtained during the breath-hold at CT simulation. Consequently, subsequent fractions depend on reproducing this reference geometry as closely as possible. Differences in inspiratory depth or internal thoracic configuration may therefore result in a dose distribution that differs from the intended distribution during treatment planning, even when the patient’s external position remains clinically acceptable. Online adaptive radiotherapy (oART) offers a conceptually different approach. Rather than relying exclusively on reproducing the simulation anatomy, volumetric imaging obtained immediately before treatment can evaluate the day’s anatomy, recalculate the reference plan, and when appropriate, reoptimize the treatment plan to the current target and OAR geometry. In patients treated in DIBH, this allows for adaptation of the treatment plan to the breath-hold anatomy actually achieved during a particular fraction. Thus, oART may reduce, but not eliminate, dependence on the exact reproduction of the simulated DIBH while preserving the fundamental geometric and cardiac-sparing advantages of breath-hold.

Recent advances in CBCT imaging, artificial intelligence-based segmentation, and automated treatment planning have made these workflows clinically feasible. Initial experience with the Ethos platform demonstrated the feasibility of CBCT-guided breast oART and showed improved target coverage with adapted plans compared with scheduled plans [7]. Subsequent work specifically evaluating Ethos auto-segmentation for whole-breast treatment confirmed that automated contouring can provide a clinically usable basis for adaptation, while emphasizing the continued need for physician review and correction [8]. In parallel, advances in high-quality CBCT imaging have improved the reliability of dose calculation on daily volumetric datasets. HyperSight iterative CBCT has demonstrated dose-calculation performance comparable to synthetic CT in breast and pelvic oART applications [9].

Clinical evidence for breast oART has also expanded over the past two years. In the prospective BREAST-ART implementation study, Nelissen et al. treated patients with right-sided whole-breast irradiation using 26 Gy in five fractions and demonstrated the feasibility of comparing the reference plan recalculated on the daily anatomy with a newly optimized adaptive plan within the same treatment session [10]. More recently, van Vlaenderen et al. reported broader clinical implementation across partial-breast, whole-breast, post-mastectomy, and axillary target volumes [11] Adapted plans consistently met the predefined target-coverage criteria, and acute toxicity was predominantly grade 0–1 These studies demonstrate that CBCT-guided breast oART has progressed from a technical feasibility concept toward clinical implementation. However, available evidence remains limited and has largely focused on workflow feasibility, auto-segmentation, and implementation. Data specifically evaluating the dosimetric effect of adaptation across individual fractions of ultrahypofractionated whole-breast radiotherapy, particularly when combined with daily DIBH and accompanied by clinical acute-toxicity assessment, remain scarce.

The present study assessed our clinical experience with CBCT-guided online adaptive ultrahypofractionated whole-breast radiotherapy in patients with early-stage breast cancer. By comparing the treatment plan recalculated from the patient’s treatment-day anatomy with the corresponding online-adapted plan, we aimed to quantify the impact of daily adaptation on target coverage and OAR exposure, and to evaluate acute treatment-related toxicity. We hypothesized that daily online adaptation would improve target coverage while maintaining clinically acceptable OAR doses and low acute toxicity. Furthermore, in patients undergoing DIBH, we anticipated that adaptation would reduce reliance on precise reproduction of the simulation breath-hold geometry by customizing treatment to the anatomy present at each fraction.

## 2. Materials and Methods

### 2.1. Study Design, Patient Population and Toxicity Assessment

This retrospective, single-center study included 55 patients with early-stage breast cancer treated with adjuvant CBCT-guided online adaptive whole-breast radiotherapy at the Specialty Hospital Radiochirurgia Zagreb from March 2024 to April 2026. All patients underwent breast-conserving surgery and subsequently received ultra-hypofractionated whole-breast irradiation in five fractions using an online adaptive workflow. The study population included patients with in situ or invasive breast carcinoma with pathological T categories ranging from T0 to T2 and predominantly node-negative disease; one patient had N1mi disease. The present analysis was restricted to whole-breast irradiation and did not include regional nodal target volumes. We collected clinical, pathological, treatment, dosimetric, and toxicity data retrospectively from institutional medical records and treatment-planning systems. Patient and tumor characteristics included age, tumor laterality, pathological stage, histologic grade, hormone receptor and HER2 status, Ki-67 proliferation index, and systemic therapy use.

The present analysis focused on dosimetric outcomes and acute treatment-related toxicity. We defined acute toxicity as any radiation-related adverse event occurring during radiotherapy or within 90 days after treatment completion and graded it according to the Common Terminology Criteria for Adverse Events (CTCAE), version 5.0. Late toxicity and long-term oncological outcomes were outside the scope of this analysis.

### 2.2. CT Simulation, Patient Positioning, and Respiratory Motion Management

Before computed tomography simulation, patients received respiratory coaching to establish a stable, reproducible DIBH. DIBH training was individualized rather than performed for a predefined duration. Patients first underwent a brief coaching session using the Varian Respiratory Gating for Scanners system to assess their ability to achieve and reproduce a breath hold. Patients considered suitable for DIBH subsequently received additional training at the linear accelerator using Vision RT surface guidance. If a patient was able to maintain a breath hold but showed insufficient reproducibility, additional respiratory coaching with a physiotherapist was provided over approximately 2–3 days before treatment. We evaluated the ability to achieve and sustain a consistent breath-hold using surface guidance, with additional coaching as needed. We performed a treatment-planning CT using a photon-counting CT scanner (NAEOTOM Alpha, Siemens Healthineers, Forchheim, Germany). We positioned patients supine with both arms elevated above the head and supported with an appropriate breast immobilization device. We used a wing board as the standard immobilization device when the position was comfortably tolerated and reproducible. A personalized vacuum cushion was used primarily in patients with spinal or other musculoskeletal conditions that limited tolerance or reproducibility of the standard wing-board position. We performed imaging without intravenous contrast during DIBH. The resulting photon-counting CT dataset served as the reference image set for delineating targets and organs at risk and for subsequent treatment planning. DIBH was employed to ensure a reproducible respiratory condition during simulation and to augment thoracic expansion.

### 2.3. Target Volume and Organ-at-Risk Delineation and Treatment Planning

Target volumes were delineated on the planning photon-counting CT dataset in accordance with the ESTRO consensus recommendations for elective radiotherapy of early-stage breast cancer [12]. The whole-breast clinical target volume (CTV) encompassed visible glandular breast tissue and postoperative breast parenchyma, following the anatomical boundaries defined by the ESTRO guideline. Anteriorly, the CTV was limited to approximately 5 mm beneath the skin surface, while the posterior border followed the anterior surface of the pectoral fascia, excluding the pectoral musculature, ribs, and lung. The cranial, caudal, medial, and lateral boundaries were individualized based on visible breast tissue and established anatomical landmarks. We then added a 5-mm isotropic margin to the breast CTV to generate the planning target volume (PTV), and cropped the PTV 5 mm from the external body contour anteriorly. We delineated OARs on the same planning dataset, including the ipsilateral and contralateral lungs, heart, and contralateral breast. For left-sided tumors, the left anterior descending coronary artery (LAD) was additionally delineated to enable dedicated assessment of coronary exposure.

We generated reference treatment plans in the Ethos Treatment Planning System using the FAST-Forward planning pack as a framework. We adopted planning objectives for PTV coverage and dose homogeneity, along with dose constraints for the heart and ipsilateral lung, from the FAST-Forward trial protocol [1]. The default Ethos planning workflow generated candidate plans using equidistant static-field IMRT and full-arc VMAT geometries. Because VMAT optimization and plan calculation were consistently more time-consuming and could prolong the online adaptive treatment session, we selected a sliding-window IMRT technique for clinical use. For each patient, we created an individualized, anatomy-based tangential arrangement of seven to nine fields. We managed patients treated before the platform upgrade using Ethos version 1, in which we calculated the adapted dose distribution on a synthetic CT generated through structure-guided deformable registration of the planning CT to the daily CBCT. After upgrading to Ethos version 2.0 and clinically commissioning HyperSight CBCT for direct dose calculation, including establishing and validating an appropriate CT-number-to-electron-density calibration curve, we performed dose calculation for online adaptation directly on the daily HyperSight CBCT dataset.

The prescribed whole-breast dose was either 26 Gy in five daily fractions of 5.2 Gy or 27 Gy in five daily fractions of 5.4 Gy, delivered over one week, reflecting the two ultrahypofractionated schedules investigated in FAST-Forward [1]. During the initial period of institutional implementation, we used both five-fraction schedules. After presenting the mature 10-year FAST-Forward results at the ESTRO 2025 Congress, we discontinued the 27-Gy regimen at our institution and subsequently adopted 26 Gy in five fractions as the standard ultrahypofractionated prescription. The mature FAST-Forward data confirmed sustained long-term efficacy and a late normal-tissue effect profile for 26 Gy comparable with that of 40 Gy in 15 fractions, whereas the 27-Gy schedule remained associated with a greater burden of late breast and chest-wall effects [13,14]. In two younger patients considered at higher risk of local recurrence, we delivered a simultaneous integrated boost (SIB) to the tumor bed to a total dose of 29 Gy in five fractions, while maintaining the whole-breast prescription at 26 Gy in five fractions. This approach was based on emerging clinical experience supporting the feasibility and early safety of a 29-Gy SIB delivered within a five-fraction ultrahypofractionated whole-breast regimen [15].

### 2.4. Online Adaptive Radiotherapy Workflow and Treatment Delivery

Treatment was administered utilizing a Varian Ethos linear accelerator (Varian Medical Systems, Palo Alto, CA, USA) equipped with HyperSight CBCT and an integrated online adaptive radiotherapy platform. We performed initial patient setup using the AlignRT InBore surface-guided radiation therapy system (Vision RT, London, UK). We designated a predefined region of interest for continuous surface monitoring and performed initial translational and rotational adjustments before repositioning the treatment couch to the treatment isocenter.

**Figure 1:**
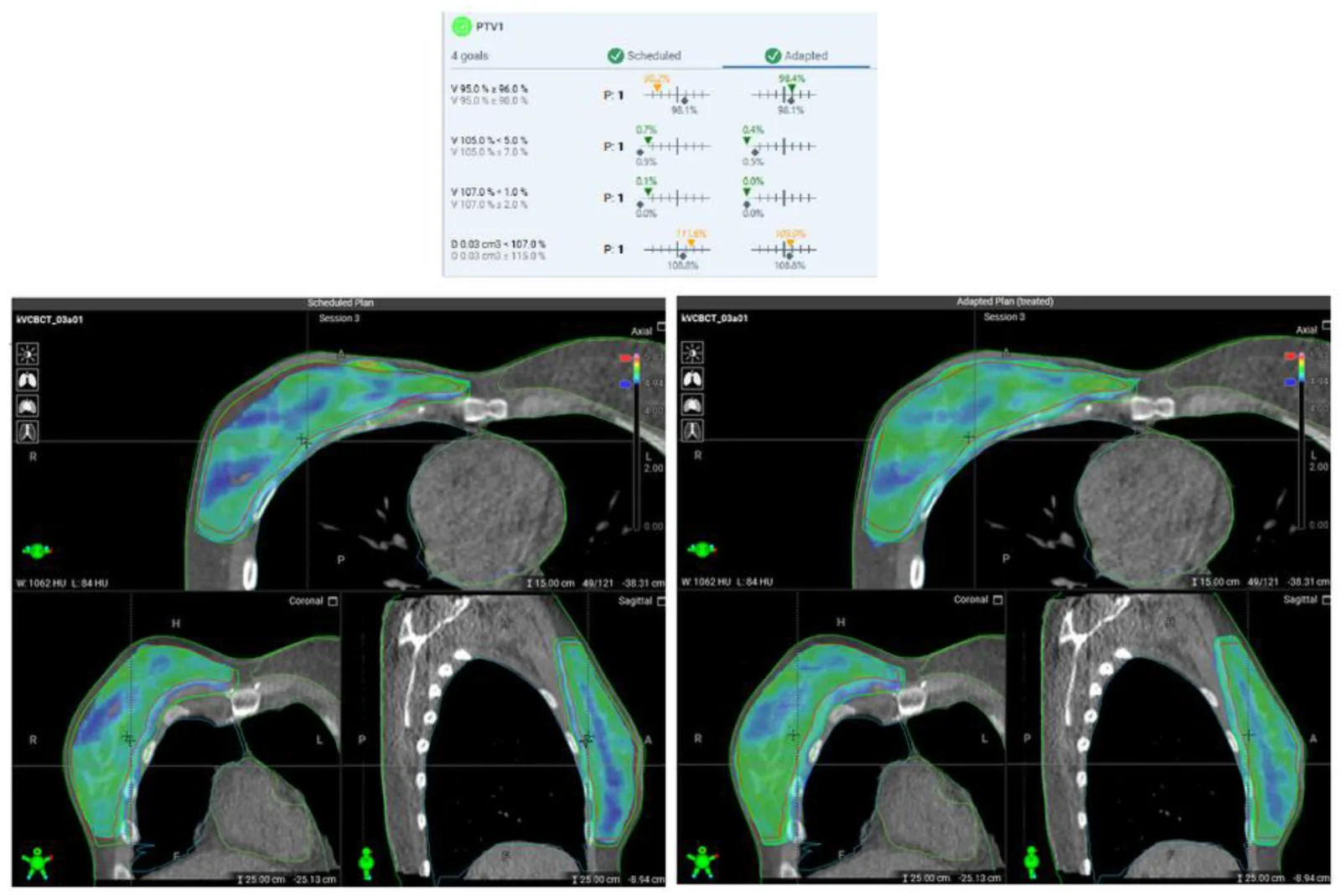
Representative example of daily online adaptation showing improved target coverage with the adapted plan (**bottom right**) compared to the scheduled plan (**bottom left**). Dashed lines represent reference lines displayed by the treatment planning system, while capital letters indicate standard anatomical orientation markers (R, right; L, left; A, anterior; P, posterior; H, head).

For each treatment fraction, we obtained a CBCT scan during DIBH. Based on the anatomy observed in the daily CBCT, the Ethos artificial intelligence-based contouring system automatically segmented organs at risk, which the treating radiation oncologist subsequently reviewed, and if necessary, modified. The daily clinical target volume was also assessed and adjusted based on the day’s anatomy. The system then generated two dose distributions: the scheduled plan, the original treatment plan recalculated on the daily anatomy, and the adapted plan, reoptimized based on the daily target and organ-at-risk geometry. We evaluated both plans against predefined target coverage and organ-at-risk dose constraints and selected the superior plan for delivery (Figure 1).

Significantly, the online adaptive workflow permitted treatment planning to be tailored to the DIBH anatomy achieved by the patient on each treatment day. In a conventional non-adaptive DIBH workflow, the treatment plan is based on the anatomy acquired at the simulation stage, and treatment delivery relies on reproducing the simulation’s breath-hold geometry as closely as possible. Conversely, online adaptation reduces this dependence by reoptimizing the treatment plan to match the patient’s actual daily breath-hold anatomy.

**Figure 2:**
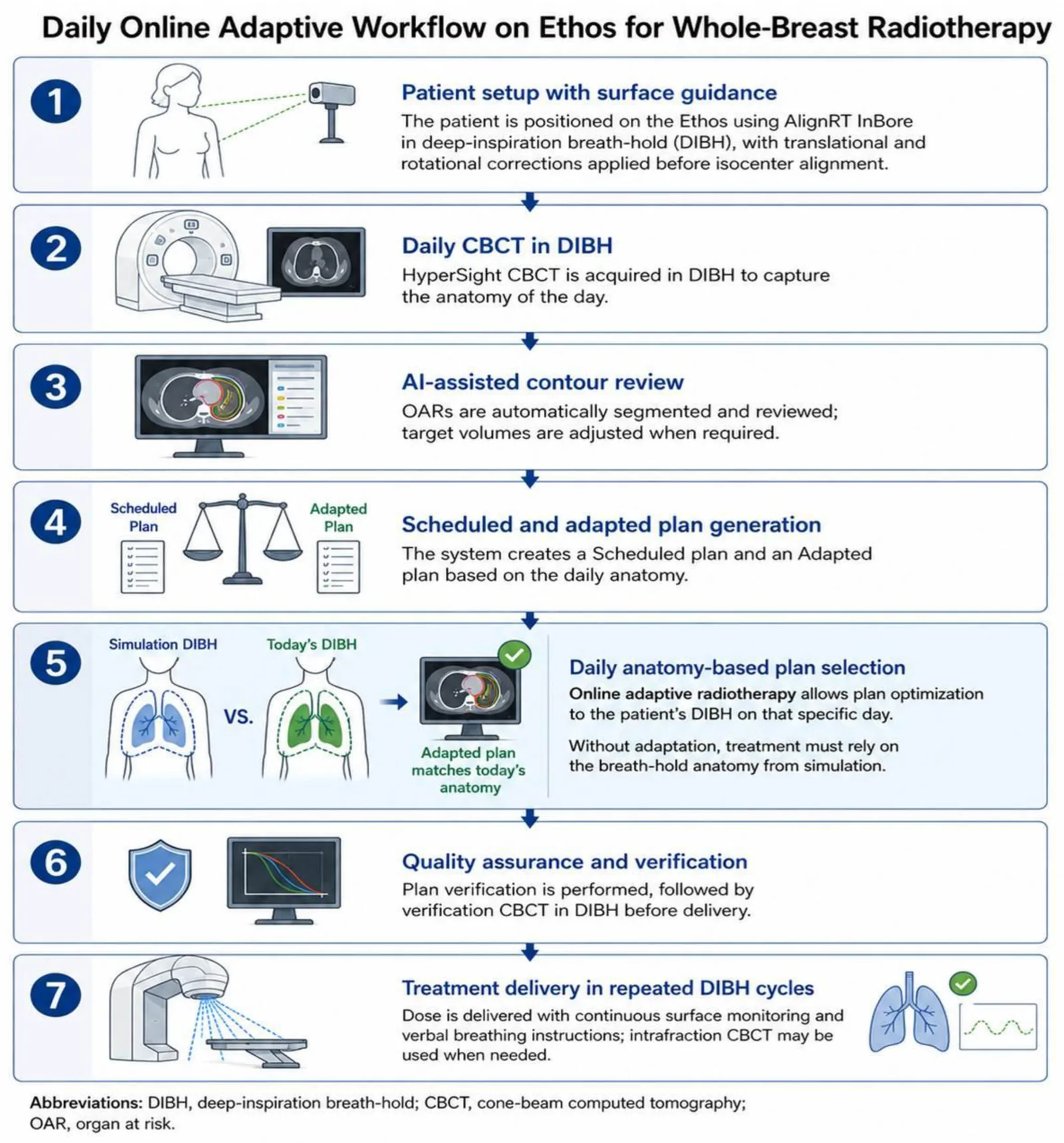
Schematic overview of the daily CBCT-guided online adaptive radiotherapy workflow on the Ethos platform, from surface-guided DIBH patient setup and daily imaging to contour review, plan adaptation, verification, and treatment delivery.

Before treatment, we performed plan quality assurance using the integrated Ethos verification workflow, complemented by an independent secondary dose calculation using Mobius3D. After the radiation oncologist and medical physicist approved the selected treatment plan, we performed an additional verification CBCT scan during DIBH to confirm patient positioning and anatomical stability before beam delivery. Treatment was administered during repeated DIBH cycles, with continuous surface guidance and verbal breathing instructions maintained throughout to ensure reproducibility of breath-hold and patient positioning. When clinically indicated, an additional intrafraction CBCT scan was performed as an additional quality-control measure to assess anatomical stability during the treatment session (Figure 2).

### 2.5. Statistical Analysis

Statistical analyses were performed using Python version 3.13.5 (Python Software Foundation, Wilmington, DE, USA). The analysis included descriptive methods and linear mixed-effects modeling. We summarized continuous variables using measures of central tendency and dispersion, including the mean, standard deviation, median, and interquartile range, as appropriate. We presented categorical variables as frequencies and percentages. To account for the non-independence of repeated treatment fractions within the same patient, all dosimetric comparisons between the scheduled and adapted treatment plans were performed using linear mixed-effects models (LMMs), with treatment plan (scheduled vs. adapted) included as a fixed effect and patient as a random intercept. The intraclass correlation coefficient (ICC) was calculated for each dosimetric endpoint to quantify the proportion of total variance attributable to between-patient differences. Cardiac dosimetric assessments were confined to patients with left-sided breast cancer for whom paired measurements were available. All statistical tests were two-sided, and *p*-values < 0.05 were considered statistically significant.

## 3. Results

The study cohort comprised 55 patients with early-stage breast cancer (Table 1), with a median age of 57 years (range, 27–74 years). Most patients had node-negative disease (83.6%) and T1 tumors (63.6%), while 83.6% and 80.0% were estrogen- and progesterone-receptor positive, respectively; 34 patients (61.8%) had right-sided and 21 (38.2%) left-sided breast cancer.

Since comprehensive dosimetric datasets were accessible for 52 out of the 55 patients within the clinical cohort, the dosimetric analysis encompassed these individuals. These patients underwent 260 treatment fractions, with paired scheduled and adapted plan data available for 259 fractions. The adapted plan was implemented for 242 out of the 260 fractions (93.1%), whereas the scheduled plan was used for 18 fractions (6.9%), indicating that daily reoptimization was dosimetrically advantageous in most treatment sessions. The mean treatment-session duration, measured from acquisition of the first CBCT to completion of dose delivery, was 28 min per fraction.

**Figure 3:**
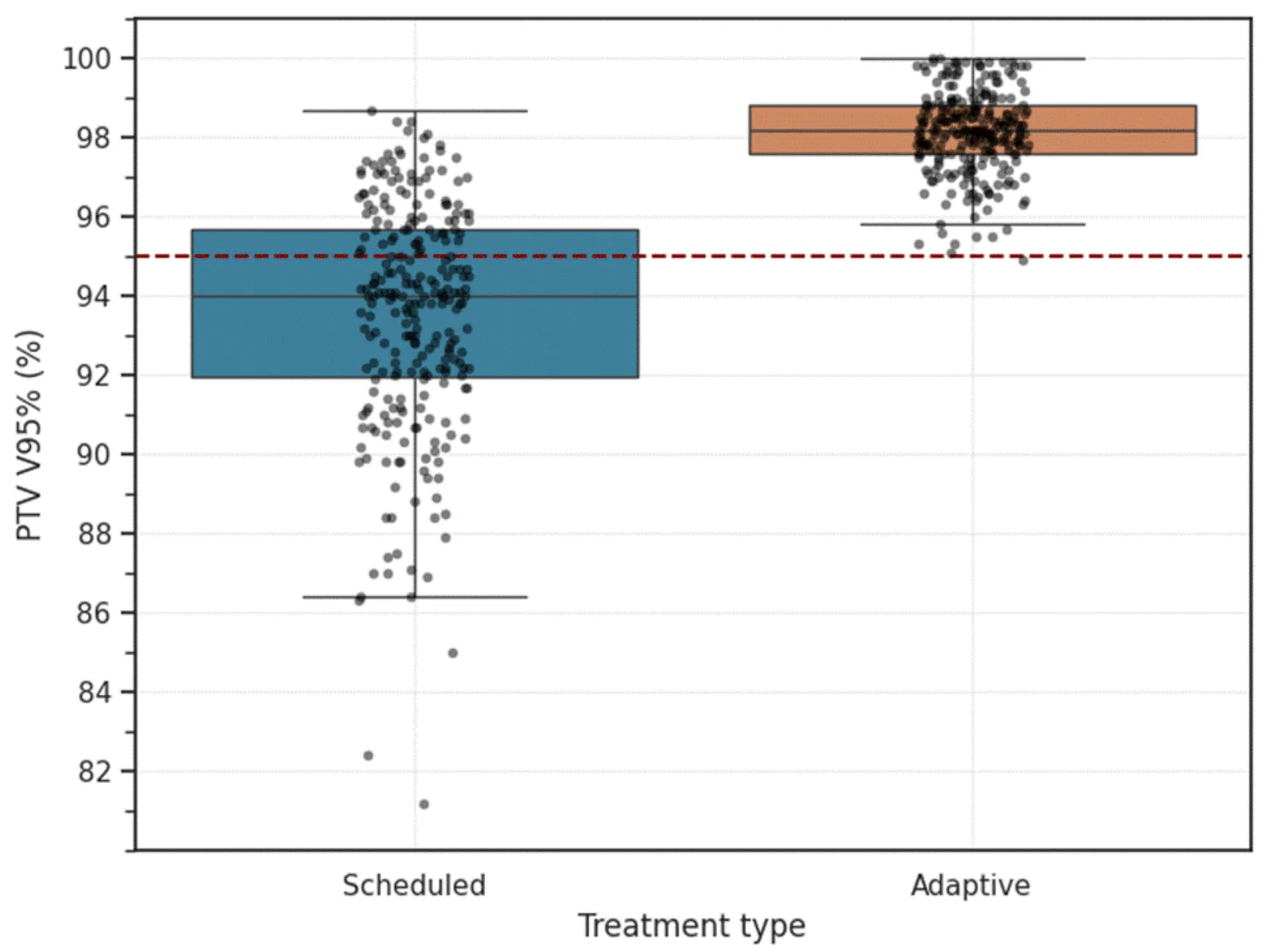
Comparison of planning target volume coverage between the scheduled and online-adapted treatment plans. Box plots represent PTV V95% (%) across evaluable treatment fractions, with individual points representing single fractions. The red dashed horizontal line indicates the reference threshold of 95% for PTV V95%.

The principal dosimetric benefit of online adaptation was observed in target coverage. Across the 259 paired fractions, mean PTV V95 increased from 93.52 ± 2.94% with the scheduled plan to 98.13 ± 1.04% with the adapted plan. The corresponding median values were 94.0% (IQR, 91.95–95.70%) and 98.2% (IQR, 97.60–98.80%), respectively. The linear mixed-effects model confirmed a highly statistically significant improvement in PTV V95% with the adapted plan (*p* < 0.001). The ICC for PTV V95% was 0.347. In addition to increasing overall coverage, adaptation substantially reduced PTV V95% variability, as reflected in the narrower interquartile range and standard deviation of the adapted plans. Scheduled-plan PTV V95% ranged from 81.2% to 98.7%, whereas adapted-plan values ranged from 94.9% to 100.0% (Figure 3). Thus, while some scheduled plans showed clinically relevant undercoverage when recalculated on the daily anatomy, adaptation shifted the distribution toward consistently high target coverage.

**Figure 4:**
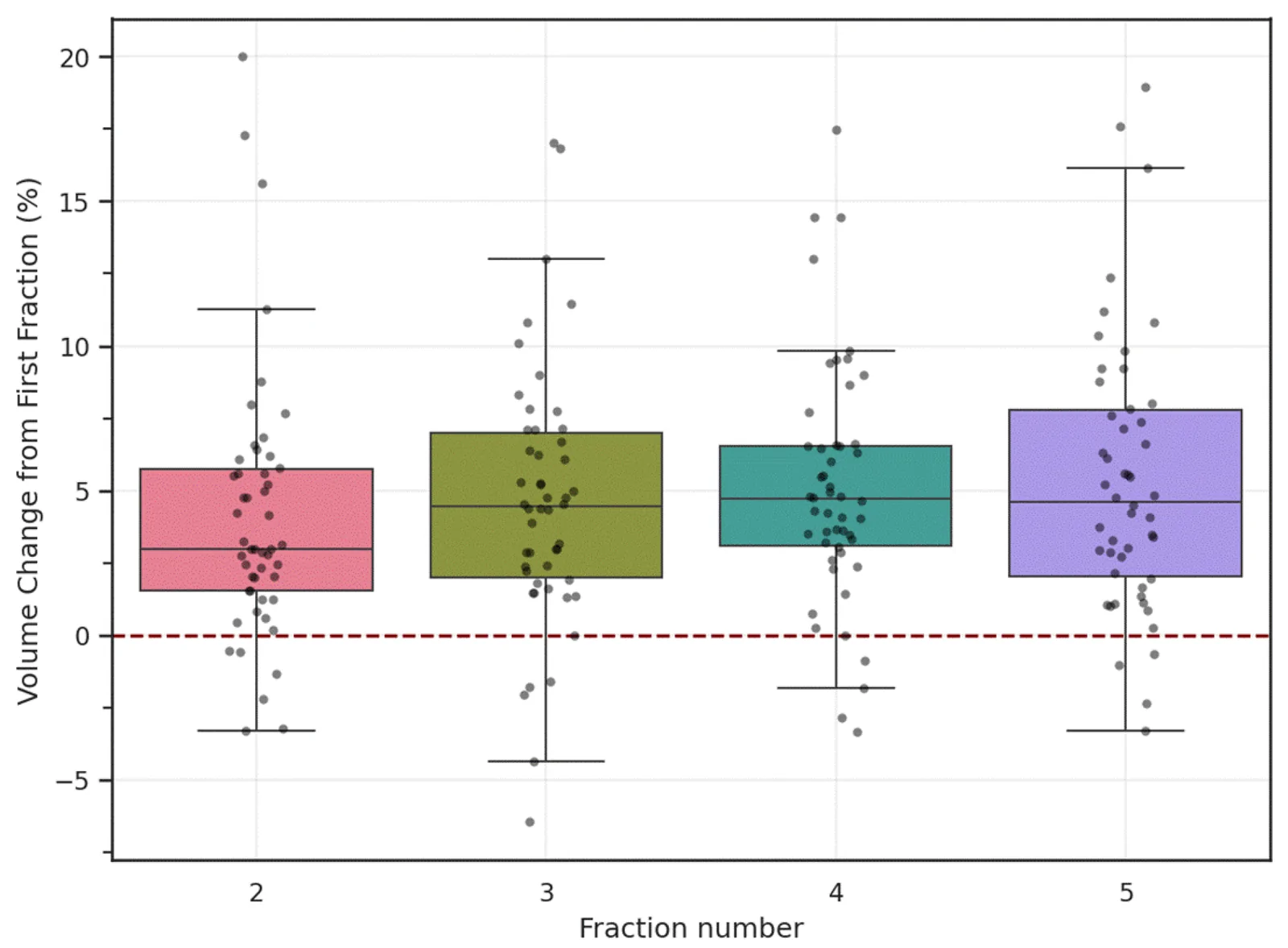
Interfraction variation in planning target volume during the five-fraction treatment course. Relative PTV volume changes during fractions 2–5 are expressed as percentage change from the PTV volume measured at the first treatment fraction. Individual points represent treatment fractions, and the dashed horizontal line indicates no change relative to fraction 1.

Daily volumetric imaging also demonstrated that breast target geometry was not static during the five-fraction treatment course. Relative changes in PTV volume compared with the first fraction were observed during fractions 2–5, with both increases and decreases despite standardized immobilization, DIBH, and surface-guided positioning. Median volume changes remained positive across the subsequent fractions, with substantial interpatient and interfraction variability; in selected fractions, increases approached 20% relative to fraction 1 (Figure 4).

**Figure 5:**
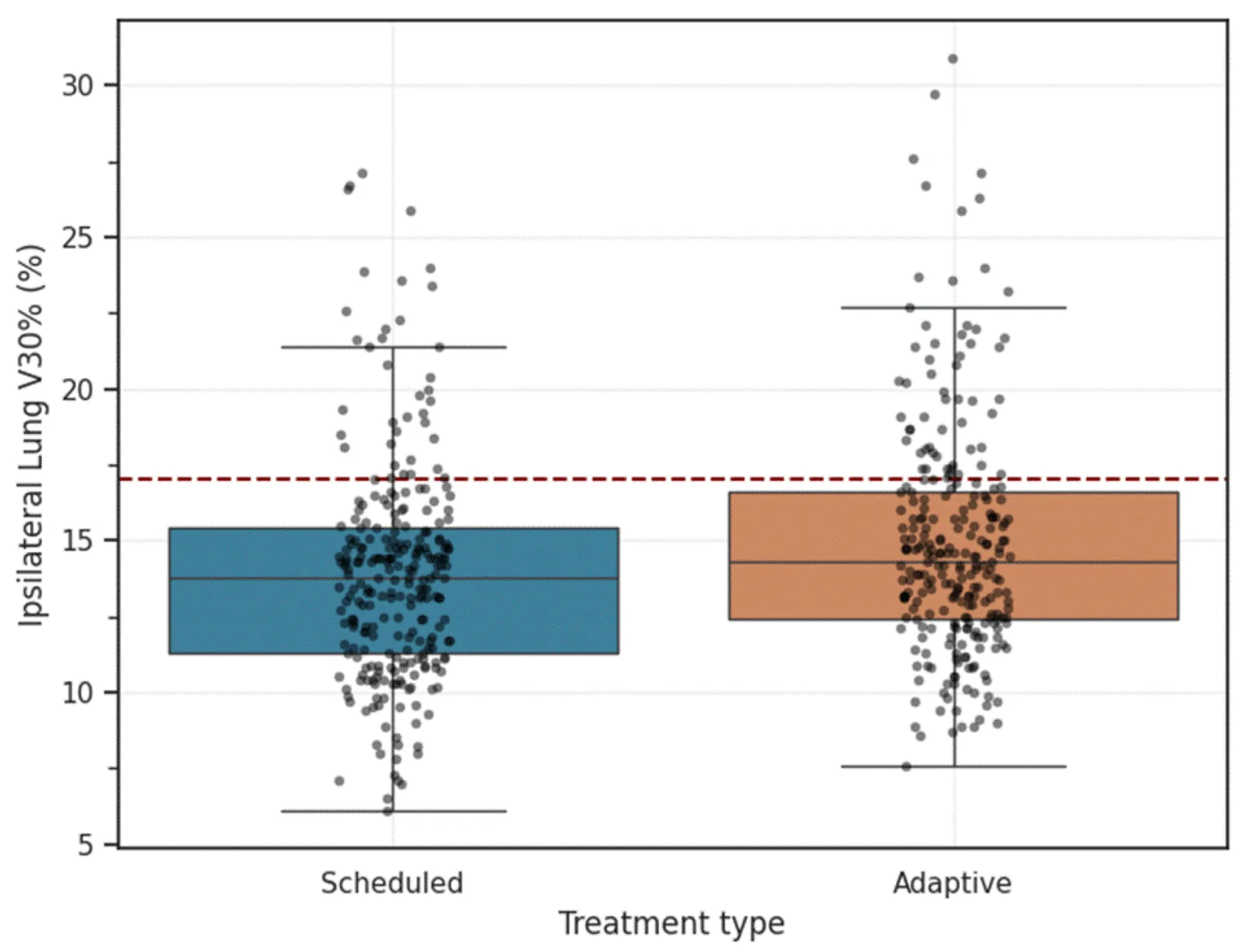
Comparison of ipsilateral lung V30 between the scheduled and online-adapted treatment plans. Individual points represent treatment fractions, and the dashed horizontal line indicates the predefined planning constraint of V30% = 17%.

For the ipsilateral lung, mean V30 increased from 13.94% in the scheduled plans to 14.96% in the adapted plans. Although the absolute difference was modest, the linear mixed-effects model confirmed a statistically significant increase in ipsilateral lung V30 with the adapted plan (*p* < 0.001). The ICC for ipsilateral lung V30% was 0.694. The FAST-Forward-based V30 constraint of 17% was met in 221 out of 259 scheduled plans (85.3%) and in 203 out of 260 adapted plans (78.1%); correspondingly, the constraint was exceeded in 38 scheduled fractions (14.7%) and 59 adapted fractions (22.7%) (Figure 5). Importantly, plan selection was based on the overall balance between target coverage and organ-at-risk sparing rather than on a single dosimetric parameter. In seven fractions, both the scheduled and adapted plans achieved PTV V95% > 95%, but only the scheduled plan met the ipsilateral lung V30% constraint; in these instances, the scheduled plan was selected for treatment delivery. At the patient level, five patients showed persistent violation of the ipsilateral lung V30% constraint across all five fractions with scheduled plans and four patients with adapted plans, with three patients in both groups. In these cases, when the lung constraint could not be consistently satisfied, the daily treatment-plan decision was primarily guided by target coverage and the overall dosimetric balance. Thus, although adaptation was associated with a small increase in ipsilateral lung exposure on a fraction-by-fraction basis, it did not translate into more patients with persistent lung-constraint violation across the entire treatment course.

**Figure 6:**
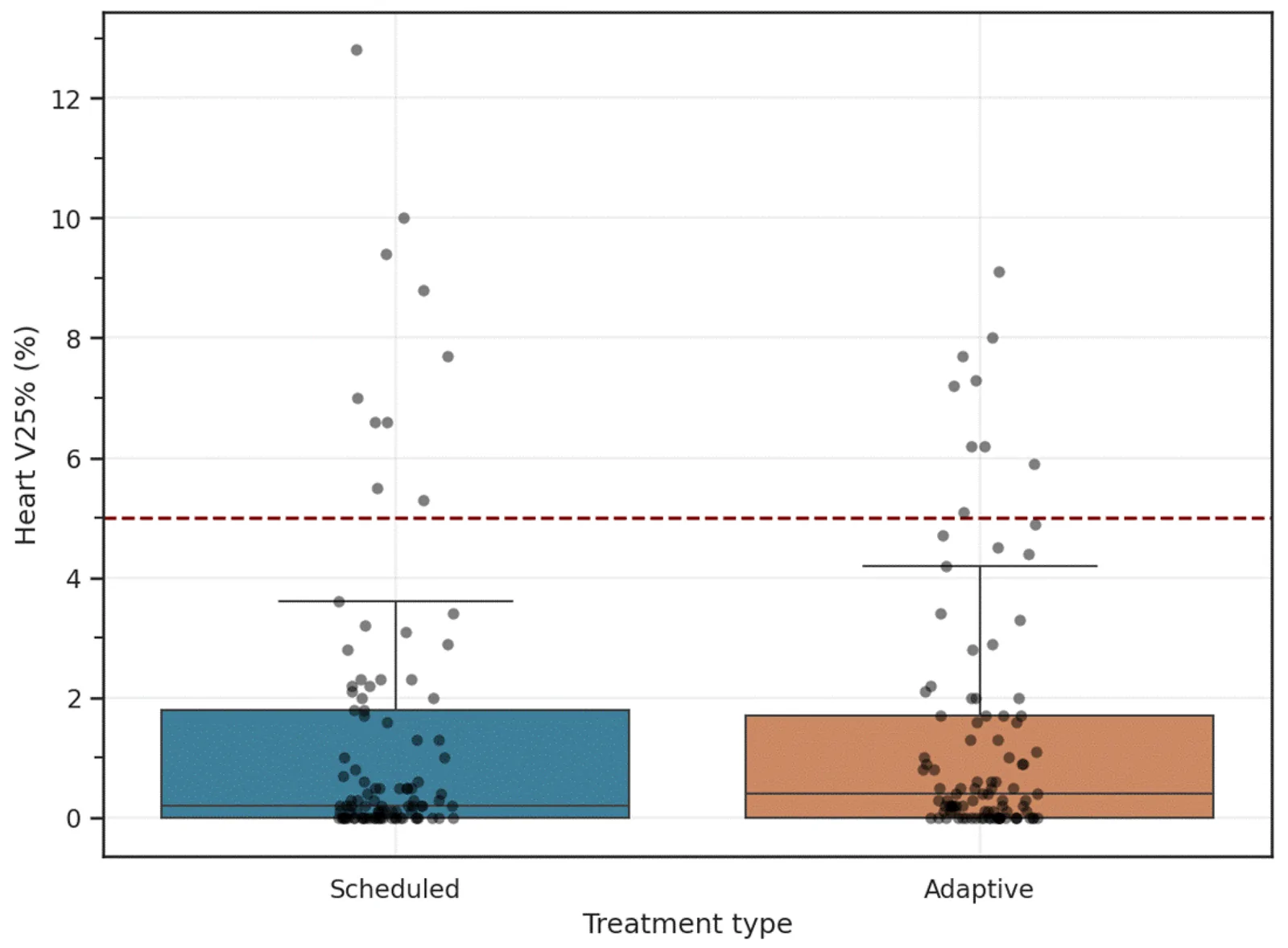
Comparison of heart V25 between the scheduled and online-adapted plans in patients with left-sided breast cancer. Individual points represent evaluable treatment fractions. The red dashed horizontal line indicates the reference dose constraint for the heart (V25Gy ≤ 5%).

Cardiac dosimetry was evaluated separately in patients with left-sided breast cancer. Heart V25% data were available for 20 patients and 99 evaluable treatment fractions. Mean heart V25% was nearly identical between the two approaches, measuring 1.400% with the scheduled plan and 1.395% with the adapted plan; median values were 0.2% and 0.4%, respectively. The linear mixed-effects model showed no statistically significant difference between the scheduled and adapted plans (*p* = 0.3825). The ICC for heart V25% was 0.827. These findings indicate that improved target coverage was not associated with a measurable increase in heart V25% (Figure 6).

**Figure 7:**
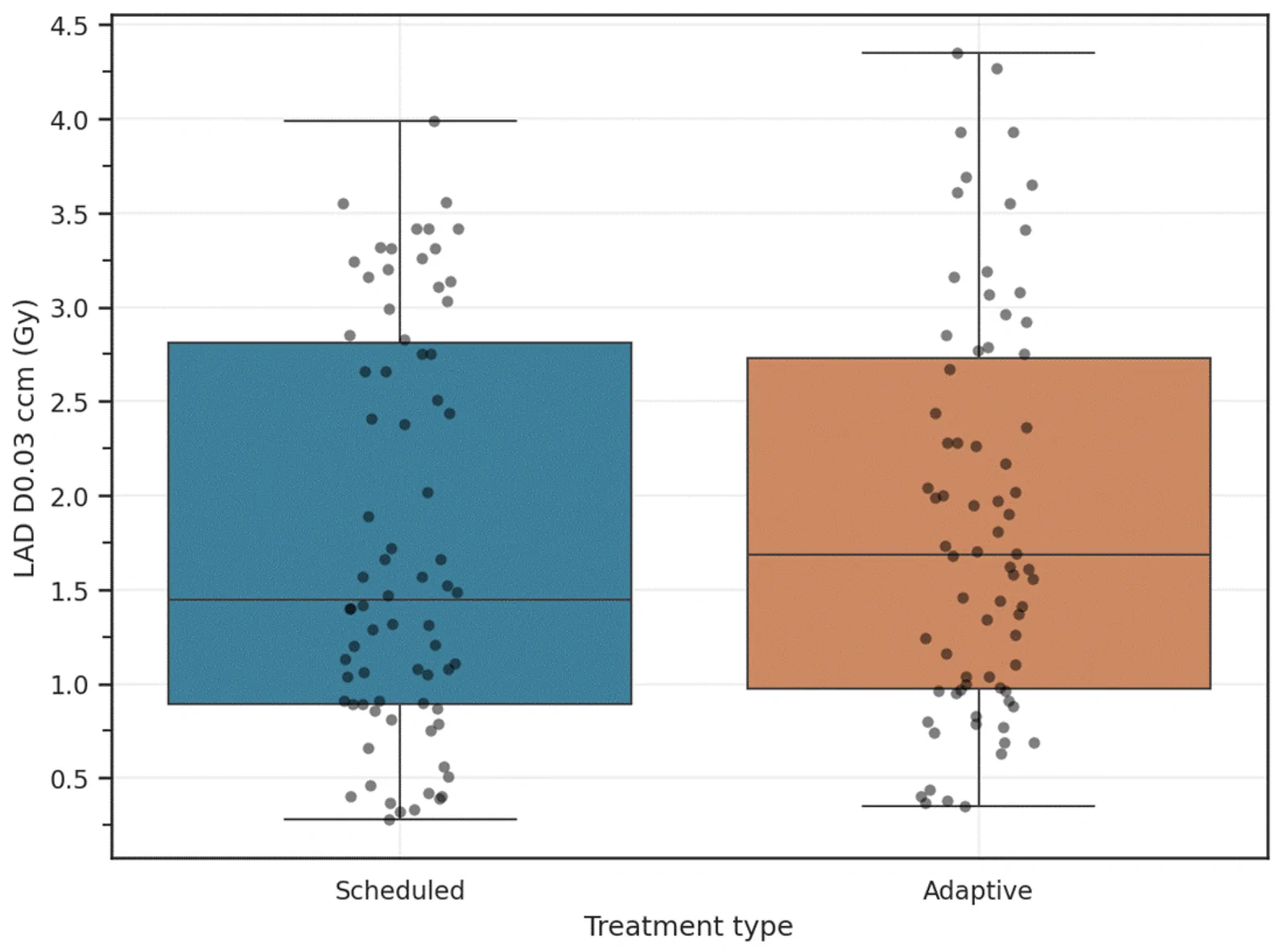
Comparison of left anterior descending coronary artery near-maximum dose between the scheduled and online-adapted plans in patients with left-sided breast cancer.

A dedicated analysis of the left anterior descending coronary artery was possible in 15 patients with left-sided breast cancer, comprising 74 paired treatment fractions. Mean LAD D0.03 cm^3^ was 1.771 Gy with the scheduled plan and 1.873 Gy with the adapted plan, while the corresponding median values were 1.445 Gy and 1.685 Gy. Although adaptation yielded numerically higher values, the linear mixed-effects model confirmed that the difference was not statistically significant (*p* = 0.1390). The ICC for LAD D0.03 cm^3^ was 0.845. (Figure 7).

The median follow-up after the completion of oART was 11.9 months (range, 0.2–27.6 months), with follow-up censored as of 1 July 2026. This study analyzed only acute radiation-related toxicity. Acute toxicity was defined as any treatment-related adverse event occurring during radiotherapy or within 90 days after the completion of treatment and was graded according to CTCAE version 5.0. Acute toxicity was observed in five patients (9.1%); two patients (3.6%) experienced grade 1 breast pain, while three patients (5.5%) developed grade 1 erythema. No grade ≥ 2 acute toxicity was observed.

## 4. Discussion

The principal finding of this study is the magnitude and consistency of improvement in whole-breast target coverage with daily online adaptation. Across the 259 paired fractions, mean PTV V95% increased from 93.52% with the scheduled plan to 98.13% with the adapted plan, and the adapted plan’s PTV coverage distribution was substantially narrower. The adapted plan was selected for treatment in 93.1% of fractions. These findings extend the still limited but rapidly evolving clinical experience with breast oART. Early Ethos studies established technical feasibility, auto-segmentation performance, and the potential for improved target coverage [7,8], while BREAST-ART subsequently demonstrated prospective clinical implementation of five-fraction whole-breast oART [10]. More recently, van Vlaenderen et al. expanded this concept to partial-breast, whole-breast, post-mastectomy, and axillary target volumes [11]. A broader review by De-Colle et al. similarly emphasized that breast adaptation may be particularly relevant when daily changes in breast shape, surgical cavity, arm position, or regional nodal geometry challenge the reference plan’s robustness [16]. Adaptive breast radiotherapy is also being explored on MR-guided platforms; early prone partial-breast experience and the phase II PARLOB study demonstrated that daily adaptation can be integrated into breast treatment using a different imaging and planning paradigm [17,18]. Although these populations and target concepts differ from ours, together, they show that breast oART is moving beyond proof-of-concept toward clinically implemented, anatomy-driven treatment strategies.

Adequate target coverage remains a fundamental objective of breast radiotherapy, irrespective of the planning technique. Conventional tangential fields and field-in-field approaches remain robust and widely used, whereas inverse-planned IMRT, VMAT, tangential VMAT, and hybrid strategies may improve homogeneity or conformity in selected anatomies, often at the cost of increased low-dose exposure to the lungs, heart, and contralateral tissues. Rayn et al. reported mean breast CTV and PTV coverage of 97.9% and 95.7%, respectively, using a multi-field IMRT approach developed for Halcyon/Ethos, with improved coverage of several targets compared with a hybrid 3D technique [19]. In a whole-breast study using complementary SGRT and CBCT setup, Mankinen et al. reported median CTV V95 values of approximately 98% with both tangential VMAT and 3D-CRT, although a 5-mm CTV-to-PTV margin encompassed the observed CTV positional and shape variation in only 85% of fractions [20]. Contemporary planning comparisons also illustrate that technique labels alone do not determine plan quality. Zhang et al. demonstrated different target and OAR trade-offs between hybrid IMRT and VMAT [21], while Mottareale et al. showed that arc geometry and how a hybrid strategy is implemented may matter as much as whether a plan is nominally classified as VMAT or hybrid [22]. Our findings add a different dimension to this literature. The reference plan can be technically excellent, but if it is applied to an anatomy that differs from simulation, treatment-day coverage may still be suboptimal. Online adaptation therefore does not replace careful planning technique selection; rather, it adds a mechanism to preserve the intended dosimetric quality when anatomy changes.

Whether more consistent geometric coverage ultimately translates into fewer recurrences is difficult to establish. The recently published prospective validation of the ESTRO target volume guideline from the DBCG Skagen Trial 1 is particularly informative. Among the 2963 enrolled patients, 78 developed a locoregional recurrence, and 74 of these events (95%) occurred within ESTRO-defined CTVs planned to receive the full prescribed dose; all local recurrences were within the breast or chest-wall CTV [23]. This is consistent with an earlier multi-institutional mapping study by Chang et al., in which the ESTRO CTV encompassed 97.6% of mapped locoregional recurrence sites [24]. These observations support the adequacy of contemporary anatomical target definitions and suggest that most failures are not due to geographic omission of the recommended CTV. Accordingly, our dosimetric results should not be interpreted as evidence that oART will reduce recurrence. However, accurate delineation and adequate delivered dose are separate requirements: a correctly defined CTV cannot benefit from its intended prescription if daily anatomical changes lead to systematic or repeated undercoverage. In this context, adaptation is best viewed as maintaining the planned therapeutic intent on the anatomy of the day. Demonstrating whether this translates into improved local control will require substantially larger cohorts, cumulative dose reconstruction, and long-term outcome data.

The interaction between DIBH and online adaptation is particularly relevant to the present workflow. DIBH is a well-established cardiac-sparing strategy for left-sided breast irradiation, and both meta-analytic data and contemporary surface-guided prospective experience confirm meaningful reductions in heart and LAD exposure compared with free breathing [25,26]. Current ESTRO-ACROP recommendations emphasize structured coaching, reproducible breath-hold levels, and appropriate monitoring and verification [4], while the ESTRO-ACROP SGRT guideline supports optical surface imaging as a valuable tool for setup and intrafraction monitoring [27]. Nevertheless, external surface reproducibility does not guarantee identical internal anatomy. Wu et al. demonstrated generally good DIBH stability using real-time optical surface imaging but still identified residual setup-related dosimetric sensitivity [28], and Mankinen et al. showed that CBCT provided complementary information beyond SGRT alone [20]. In a prospective comparison of voluntary DIBH, active breathing control, and SGRT-based breath hold, Chan et al. found broadly comparable reproducibility of cumulative mean heart dose, although measurable interfraction variability remained [29]. A potential advantage of oART in this setting is that the treatment plan can be reoptimized to the internal anatomy achieved during the breath hold of the individual fraction, thereby reducing reliance on exact reproduction of the simulation breath-hold geometry. The benefit of DIBH itself is heterogeneous and depends on patient anatomy, target extent, and treatment technique. Gaál et al. demonstrated substantial interpatient variation in cardiac benefit [30], while Zhou et al. proposed a free-breathing mean heart dose threshold to help identify patients most likely to benefit from DIBH [31]; the SAVE-HEART study further confirmed meaningful reductions in cardiac and coronary exposure with surface-guided DIBH [26]. These findings support an individualized approach to respiratory management. In patients selected for DIBH, daily adaptation may be particularly relevant when treatment-day internal anatomy differs from the simulation geometry, especially in the presence of borderline heart or lung geometry or complex target–OAR relationships. The gain in target coverage in our cohort was accompanied by a modest increase in ipsilateral lung exposure. Mean ipsilateral lung V30% increased from 13.94% to 14.96%, and more fractions exceeded the FAST-Forward V30% planning constraint after adaptation. This finding is clinically important because adaptation should not be equated with universal improvement in every dosimetric endpoint. The adapted mean remained close to the FAST-Forward optimal objective and below the mandatory limit used in the trial planning framework [1], but restoration of peripheral breast coverage can inevitably bring the prescription isodose closer to the adjacent lung. Pulmonary dose should therefore remain an active competing objective rather than being relaxed simply to maximize PTV coverage. Importantly, the relative-dose V30% metric used in FAST-Forward is not numerically interchangeable with the absolute-dose V20 Gy or V30 Gy metrics reported in other fractionation schedules. In conventional breast radiotherapy, Karlsen et al. found V20 Gy, V30 Gy, and mean lung dose to be associated with radiological pneumonitis [32]. More recently, Ward et al. evaluated five-fraction breast radiotherapy and observed grade ≥2 pneumonitis in 3.6% of 224 patients, with pneumonitis risk increasing at higher intermediate-to-high ipsilateral lung doses and an exploratory V20 Gy threshold associated with greater risk [33]. Systematic analyses of modern breast radiotherapy also emphasize that lung dose and long-term risk depend on the entire dose distribution and patient-related factors, not a single DVH point [34,35]. Thus, the approximately one-percentage-point increase in our relative V30 cannot be translated directly into a quantified clinical risk, but it reinforces the need to preserve OAR constraints during adaptation and to evaluate cumulative delivered dose in future studies.

Heart V25% was essentially unchanged between the scheduled and adapted plans, and the numerical increase in LAD D0.03 cm^3^ did not reach statistical significance. This matters because even small differences in cardiac exposure may matter over long-term survivorship. Darby et al. demonstrated a linear increase in major coronary events with increasing mean heart dose, without evidence of a safe threshold [36], and modern risk analyses continue to support minimizing both cardiac and pulmonary exposure whenever achievable [34]. In our left-sided subgroup, DIBH established a low-dose cardiac geometry and online adaptation preserved target coverage without a measurable penalty in heart V25%. This supports the concept that DIBH and adaptation are complementary rather than competing strategies: breath hold provides the primary anatomical displacement of the heart, while adaptation addresses residual day-to-day geometric variation.

Early clinical tolerance was favorable, with acute radiation-related toxicity documented in only five patients (9.1%); all events were grade 1, consisting of breast pain in two patients and erythema in three, and no grade ≥ 2 toxicity was observed. These findings are consistent with the overall safety of modern five-fraction breast radiotherapy, but the absolute rate in our cohort should be interpreted cautiously. In a prospective implementation series of 26 Gy in five fractions, Nugent et al. reported grade 1 and grade 2 acute skin toxicity in 48% and 32% of evaluable patients, respectively [37,38]. The prospective real-world cohort reported by Mink van der Molen et al. similarly demonstrated acceptable acute toxicity and patient-reported outcomes with five-fraction treatment [2], while Dzhugashvili et al. observed predominantly low-grade acute toxicity in a five-fraction cohort that also included SIB treatment [15]. In BREAST-ART, no grade 3 or higher toxicity was reported [10], and van Vlaenderen et al.’s expanded adaptive experience remained predominantly grades 0–1 [11]. The substantially lower recorded rate in our cohort most likely reflects differences in retrospective versus prospective toxicity capture, assessment timing, and documentation rather than a true toxicity reduction attributable to adaptation. Therefore, our data support short-term feasibility and tolerability but do not establish a toxicity advantage over non-adaptive ultrahypofractionated breast radiotherapy. The favorable early toxicity reported in MR-guided adaptive partial-breast series [17,18] further supports the general feasibility of adaptive breast treatment, although those smaller target volumes are not directly comparable with whole-breast irradiation.

A clinically important question is which patients derive sufficient incremental benefit to justify a full online adaptive workflow. The present results suggest that adaptation is most valuable when the scheduled plan is vulnerable to daily geometric change: larger or more mobile breasts, variable arm or shoulder positioning, postoperative or oncoplastic changes, changing seroma, borderline target-OAR geometry, variable DIBH anatomy, or treatment plans incorporating a boost or more complex regional targets [11,16]. From a practical perspective, potential triggers for online adaptation could therefore include substantial treatment-day changes in breast or thoracic geometry, failure of the scheduled plan to maintain adequate PTV coverage, or proximity to predefined heart or lung dose constraints, particularly when such deviations are already evident during the first treatment fractions. Conversely, patients with stable anatomy and consistently robust scheduled-plan coverage may gain little from daily reoptimization. This distinction is important because oART has a measurable resource cost. In a large early Ethos implementation experience, Stanley et al. reported an average total adaptive session duration of approximately 35 min, with about 20 min attributable to the adaptive component, and the adaptive plan was selected in 92% of adaptive sessions across disease sites [39]. Longer sessions also increase the relevance of intra-fraction monitoring; complementary surface imaging during oART has demonstrated measurable patient movement over the course of adaptation and treatment [40]. These observations support developing selective or trigger-based breast adaptation rather than assuming that every patient requires the same degree of online replanning. Future studies should therefore aim not only to demonstrate dosimetric benefit, but also to identify pretreatment or early-fraction predictors of who is most likely to benefit.

This study has several limitations. Its retrospective, single-center design introduces the potential for selection and documentation bias and limits generalizability. The clinical cohort included 55 patients, while complete paired dosimetric data were available for 52, and cardiac analyses were restricted to smaller left-sided subsets. Dosimetric comparisons were performed at the fraction level; repeated fractions from the same patient are not fully independent, and the Wilcoxon analysis does not explicitly model this hierarchical structure. Future studies should therefore incorporate patient-level aggregation or mixed-effects approaches. We compared scheduled and adapted plans on the anatomy of the day but did not perform deformable cumulative dose accumulation, limiting conclusions regarding the total delivered OAR dose. The cohort also spans two Ethos 2.0 software generations and includes both 26-Gy and 27-Gy prescriptions, with two patients receiving a 29-Gy simultaneous integrated boost, introducing technical and treatment heterogeneity. In addition, acute toxicity was retrospectively extracted; no prospectively collected patient-reported outcomes or formal cosmetic assessments were available, and follow-up is insufficient for late toxicity or oncological outcomes. Finally, the consistent dosimetric superiority of the adapted plan does not establish that every observed percentage-point improvement is clinically meaningful. Nevertheless, the magnitude and consistency of the treatment-day coverage improvement, together with preserved cardiac dosimetry and acceptable early tolerance, support prospective studies incorporating cumulative dose, standardized toxicity and cosmesis assessment, patient-reported outcomes, and predefined criteria for selective use of breast oART.

## 5. Conclusions

CBCT-guided online adaptive ultrahypofractionated whole-breast radiotherapy consistently improved target coverage by accounting for the anatomy achieved at each treatment fraction. Importantly, this dosimetric gain was obtained while preserving cardiac sparing, with only a modest increase in ipsilateral lung exposure and a low incidence of acute toxicity. Within a DIBH-based workflow, the principal value of online adaptation may therefore extend beyond conventional image guidance by reducing dependence on exact reproduction of the simulation breath-hold geometry and allowing reoptimization to the patient’s actual daily anatomy. The mean treatment-session duration of 28 min further supports the clinical feasibility of this approach. These findings support oART as a promising strategy for improving the geometric robustness of five-fraction whole-breast radiotherapy. However, prospective studies with larger cohorts, longer follow-up, and appropriate non-adaptive comparators are required to determine which patients derive the greatest clinical benefit and whether improved daily dosimetry translates into better long-term outcomes.

## Figures and Tables

**Table 1:** Patient, tumor, and treatment characteristics of the study cohort (*n* = 55).

Characteristic	Value
Age, years, median (range)	57 (27–74)
Laterality	
Right	34 (61.8%)
Left	21 (38.2%)
Pathologic T category	
T0	5 (9.1%)
Tis	8 (14.5%)
T1a	2 (3.6%)
T1b	17 (30.9%)
T1c	16 (29.1%)
T2	7 (12.7%)
Pathologic N category	
N0	46 (83.6%)
N1mi	1 (1.8%)
Not reported/not applicable	8 (14.5%)
Histologic grade	
Grade 1	12 (21.8%)
Grade 2	32 (58.2%)
Grade 3	11 (20.0%)
Estrogen receptor (ER) status	
Positive	46 (83.6%)
Negative	9 (16.4%)
Progesterone receptor (PR) status	
Positive	44 (80.0%)
Negative	11 (20.0%)
HER2 assessment	
Reported as negative	36 (65.5%)
IHC 1+	4 (7.3%)
IHC 2+	1 (1.8%)
IHC 3+	7 (12.7%)
Not assessed	7 (12.7%)
Ki-67 index	
<21%	18 (32.7%)
21–50%	22 (40.0%)
>50%	6 (10.9%)
Not available/not interpretable	9 (16.4%)
Systemic therapy	
Chemotherapy, yes	14 (25.5%)
Chemotherapy, no	41 (74.5%)
Endocrine therapy, yes	46 (83.6%)
Endocrine therapy, no	9 (16.4%)
Radiotherapy regimen	
26 Gy in 5 fractions	37 (67.3%)
27 Gy in 5 fractions	18 (32.7%)
Boost to 29 Gy	2 (3.6%)

Data are presented as *n* (%) unless otherwise indicated. HER2, human epidermal growth factor receptor 2; IHC, immunohistochemistry. The boost subgroup is included within the 26 Gy in 5 fractions group.

## Data Availability

The original contributions presented in this study are included in the article. Further inquiries can be directed to the corresponding author.

## Abbreviations

The following abbreviations are used in this manuscript:

3D-CRT	Three-dimensional conformal radiotherapy
ACROP	Advisory Committee in Radiation Oncology Practice
CBCT	Cone-beam computed tomography
CT	Computed tomography
CTCAE	Common Terminology Criteria for Adverse Events
CTV	Clinical target volume
DBCG	Danish Breast Cancer Group
DIBH	Deep-inspiration breath-hold
DVH	Dose–volume histogram
ESTRO	European Society for Radiotherapy and Oncology
HER2	Human epidermal growth factor receptor 2
IHC	Immunohistochemistry
IMRT	Intensity-modulated radiotherapy
LAD	Left anterior descending coronary artery
MR	Magnetic resonance
OAR	Organ at risk
oART	Online adaptive radiotherapy
PTV	Planning target volume
SGRT	Surface-guided radiation therapy
SIB	Simultaneous integrated boost
VMAT	Volumetric modulated arc therapy
